# Unlocking Intracellular Oncology Targets: The Unique Role of Antibody-Based T-Cell Receptor Mimic (TCRm) Therapeutics in T-Cell Engagers (TCEs) and Antibody-Drug Conjugates (ADCs)

**DOI:** 10.3390/cancers16223776

**Published:** 2024-11-08

**Authors:** Jeffrey Molldrem, Dongxing Zha

**Affiliations:** 1 Department of Hematopoietic Biology and Malignancy, The University of Texas M.D. Anderson Cancer Center, Houston, TX 77030, USA; 2 Department of Stem Cell Transplantation and Cellular Therapy, The University of Texas M.D. Anderson Cancer Center, Houston, TX 77030, USA; 3 Alloy Therapeutics, 275 2nd Avenue, Waltham, MA 02451, USA

**Keywords:** anti-cancers, TCRm antibodies, T cell engager, ADC, TCR therapy

## Abstract

Effectively targeting intracellular tumor-associated proteins presents a formidable challenge in oncology, as they are traditionally considered inaccessible to conventional antibody-based therapies and CAR-T cell therapies. However, recent advancements in antibody engineering have revolutionized this field, offering promising new strategies to combat cancer. This review focuses on the innovative use of T-cell receptor mimic (TCRm) antibodies within the therapeutic frameworks of T-cell engagers (TCE) and antibody-drug conjugates (ADCs). TCRm antibodies, designed to recognize peptide-MHC complexes rather than cell surface proteins, integrate the capacity of T-cells to reach intracellular targets with the unique strengths of antibodies. When incorporated into T-cell engaging therapeutics, TCRms redirect T cells to cancer cells, facilitating direct cytotoxicity. In ADCs, TCRm antibodies deliver cytotoxic agents with highly specific targeting to cancer cells, sparing healthy tissues. Together, these antibody-based strategies represent a significant leap forward in oncology, opening new avenues for the treatment of cancers previously deemed untreatable, with other potential applications in autoimmune diseases. This review discusses the mechanisms, clinical advancements, and future prospects of these cutting-edge therapies, highlighting their potential to transform the landscape of cancer treatment.

## Simple Summary

TCR-mimics (TCRm) are designed to target intracellular tumor antigens presented by major histocompatibility complex (MHC) molecules on the surface of cancer cells. A TCRm imitates the capacity of T cells receptors (TCR) interact with peptide-MHC complexes (pMHC) to reach a broad range of intracellular targets with the unique strengths of high affinity and specificity of antibodies. This capability allows them to tackle previously “undruggable” targets for therapeutics when TCRm is engineered into different mechanisms of action like TCE or ADC.

## 1. Introduction

Over the last decade, immunotherapies have redefined possibilities in oncology treatment, leveraging the power of the human immune system to precisely target and eliminate cancer cells. These therapeutics depend on the mechanics of the adaptive immune response, composed broadly of antibody responses through B lymphocytes and cell-mediated immune responses through T lymphocytes. B cell biology is fundamental in the immune system’s response to pathogens, primarily through the production of antibodies that neutralize pathogens and mark them for destruction by other immune cells. Antibody therapeutics are a well-established class of immunotherapies, with over 160 antibody-based drugs currently approved to treat a range of diseases from autoimmune disorders to cancer [1].

Monoclonal antibodies (mAbs), which are designed to target disease-related cell surface antigens and soluble molecules with high specificity, are particularly efficacious as targeted cancer treatments. A deeper understanding of the role of T cells in recognizing and eliminating infected or malignant cells has paved the way for more advanced cell-based immunotherapies. The T lymphocyte, particularly its capacity for antigen-directed cytotoxicity, has become a central focus for engaging the immune system to identify and eliminate cancer cells. Basic science discoveries elucidating the molecular and cellular biology of the T cell have led to new strategies in this fight, including immune checkpoint blockade, adoptive cell therapy, and cancer vaccinology [2].

**Figure 1:**
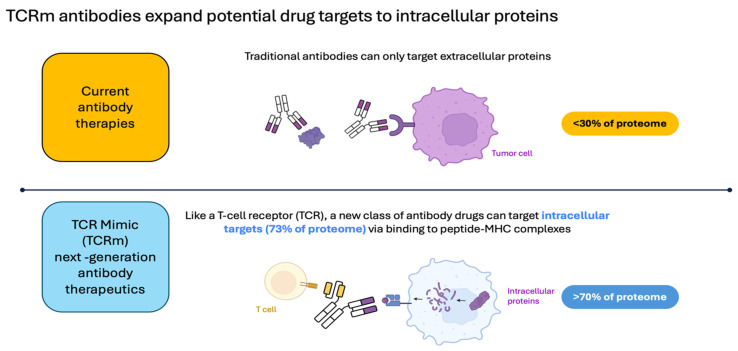
Conventional antibody therapeutics are limited to extracellular protein targets, leaving the majority of the proteome “undruggable”. TCR mimic (TCRm) antibodies, which are raised against pMHC targets and are thus able to address intracellular protein targets, introduce new therapeutic possibilities. The figure was created with BioRender.com.

As of June 2024, the U.S. Food and Drug Administration (FDA) has approved nearly thirty distinct cancer immunotherapies, including immune checkpoint inhibitors (ICIs), chimeric antigen receptor (CAR)-T cell therapies, T-cell engaging bispecific antibodies, and one tumor-infiltrating lymphocyte (TIL) therapy. While a growing number of mAb-based cancer therapeutics have entered the clinic, they face a common limitation: mAbs are limited to targeting cellular surface antigens, which represent only ~30% of the proteome and thus a small fraction of potential cancer-associated protein targets [3,4] (Figure 1).

CAR-T cell therapies, which harness the killing power of T cells, are another rapidly expanding class of cancer immunotherapies. CAR-T therapies employ an antibody fragment fused to T-cell-activating intracellular domains to create a population of T cells expressing tumor-specific antigen receptors, which can then locate, bind, and kill the targeted cancer cells. While CARs also exclusively target cellular surface antigens, their applicability has largely been limited to hematological malignancies. Thus far, no CAR-T cell therapy product has demonstrated adequate efficacy in solid tumors.

These limitations have increased interest in developing therapeutic approaches combining targeted cell-killing capacity with the unique biological features of the T-cell receptor (TCR). The TCR is a protein complex found on the surface of T cells, which, rather than targeting whole-protein antigens, recognizes short peptide antigens 8–11 amino acids in length. These peptides are presented on the cell surface by major histocompatibility complex class I (MHC-I) molecules, known as the human leukocyte antigen (HLA) system in humans [5]. TCRs’ affinity for peptide-MHC (pMHC) targets, which can include fragments of intracellular proteins degraded by the proteasome, unlocks a wide universe of tumor-associated targets.

However, translating this advantage into viable T cell-based therapies has presented continued challenges. The development and production of autologous cell-based therapies are time-, cost-, and labor-intensive, and the limited affinity, specificity, and solubility of TCRs have limited progress in creating effective TCR-based agents [6]. Advances in TCR engineering and novel technologies applying TCRs in other therapeutic modalities have powered progress in the development of powerful, precise immunotherapies for a wider range of tumor-associated targets.

## 2. Opportunities and Challenges in Therapeutic Application of TCRs

While TCR biology presents incredible therapeutic potential for recognizing a virtually limitless range of potential pMHC targets, it also confers significant limitations in the development of TCR-based drugs. TCRs can identify numerous pMHC antigens, including tumor-associated antigens and neoantigens, with relatively low affinity and specificity [7]. In contrast to the nanomolar affinities of Abs for their specific antigens, TCRs demonstrate micromolar binding affinities ranging from 1–100 μM for their cognate pMHC targets [8,9].

The receptors recognize a linear peptide sequence nestled within the MHC, as well as adjacent portions of the MHC itself. Because of the limited surface area of this interface, as well as the possibility of sequence similarities with other peptides, the discovery and development of TCR-based agents can be complicated by issues of cross-reactivity [10]. However, this potential for cross-reactivity is vital to T-cells’ function, as each TCR’s capacity to recognize thousands of different pMHC pairs enables the T-cell population to mount a sufficient protective response against actively evolving pathogens and oncogenic mutations [11]. Bioinformatics approaches that identify and validate off-target peptides present in healthy tissue are thus essential for developing safe and effective TCR-based immunotherapies.

TCRs can be harnessed in two broad therapeutic approaches: cell-based therapies, like TCR-Ts, and therapies that leverage TCRs in soluble forms, such as T-cell engagers. Tumor-infiltrating lymphocyte (TIL) therapy involves the collection, isolation, and expansion of T cells from a patient’s tumor sample but does not involve any genetic alteration of the endogenous TCR [12]. Adoptive cell therapies leveraging engineered TCRs include TCR-T cells and TCR-NK cells, both of which offer the high antigen sensitivity and diversity of potential targets conferred by TCRs [13,14]. Engineered TCR therapy equips activated T cells with specific receptors that target their complementary cancer antigens. This greatly enhances the personalization of treatment and provides a higher potential for positive therapeutic outcomes. Like CAR-T cell therapies, however, these highly personalized therapies require a grueling collection process and complex, expensive manufacturing steps, limiting their success and applicability.

### 2.1. TCR-T Therapy

The latest progress in TCR-T (T-cell receptor-engineered T cell) therapy has shown promising advancements, particularly in targeting solid tumors. Recent studies have demonstrated improved efficacy and safety through the development of TCRs with higher affinity and specificity for tumor antigens [15]. Innovations, such as using gene editing technologies like CRISPR/Cas9 to enhance TCR-T cells’ persistence and functionality, have also been reported. Additionally, combination therapies involving TCR-T cells and immune checkpoint inhibitors are being explored to overcome tumor immune evasion, leading to more robust and durable anti-tumor responses. Early-phase clinical trials have shown encouraging results, with some patients achieving partial or complete tumor regression [16]. In August 2024, the FDA granted approval to Tecelra (afamitresgene autoleucel), an autologous TCR-T cell therapy for the treatment of metastatic synovial sarcoma that modifies T-cells with TCRs specific to MAGE-A4. This landmark approval underscores the potential of TCR-Ts for targeted cancer treatment [17].

### 2.2. Bispecific T-Cell Engagers (BiTEs)

Leveraging TCRs in soluble drug forms is an attractive alternative to adoptive cell therapies, enabling “off-the-shelf” administration and circumventing the logistical and financial challenges associated with autologous TCR-T cells. However, the limited stability of native TCRs, as well as their generally weak affinities for pMHC targets, hinders their direct applicability as soluble drugs [18]. Drug developers have thus sought to recruit and redirect T cells to kill target cancer cells using T-cell engagers (TCEs). Bispecific TCEs, or BiTEs, make up the current majority of these therapies. BiTEs consist of one antibody fragment that binds to a tumor-associated antigen and another that binds to the T-cell CD3 receptor, TCEs work by bringing T cells into proximity with cancer cells and initiating their cytotoxic activity [19].

Several bispecific TCE therapies are currently approved by the FDA and the European Medicines Agency. The first, blinatumomab, was approved in the US in December 2014 as a second-line treatment for relapsed or refractory B-cell precursor acute lymphoblastic leukemia. It consists of a CD3 binding site for T cells and a CD19 binding site to target the cancer-associated B cells [20]. Other approved CD3 TCEs for hematological malignancies include talquetamab, directed to GPRC5D for the treatment of multiple myeloma, mosunetuzumab, and glofitamab, CD20-directed TCEs indicated for follicular lymphoma and large B-cell lymphoma, and CD3-BCMA approvals, e.g., Elranatamab and teclistamab, respectively [21]. More recent milestone BiTE approvals include solid tumor indications. Tarlatamab gained accelerated approval in early 2024 as a first-in-class treatment for small-cell lung cancer (SCLC) with disease progression on or after platinum-based chemotherapy [22]. Tarlatamab binds CD3 and delta-like ligand 3 (DLL3), a protein limited to the cytoplasm of healthy cells but expressed on the tumor cell surface of 85–94% of SCLCs. In a phase 2 trial of the drug, 40% of patients responded to treatment and had a median overall survival of 14.3 months, compared to typical SCLC overall survival of 8 months or fewer [23]. Tarlatamab’s safety and efficacy results in a solid tumor indication open the door to developing additional TCEs for solid tumors. This application has previously proven difficult, largely due to the lack of identified “clean” tumor-associated targets with limited expression in healthy tissues.

### 2.3. ImmTACs

While BiTEs successfully harness the cytotoxic function of T cells, their target identification and binding actions are mediated using single-chain fragment variable (scFv) antibody formats rather than TCRs. To enhance the stability and affinity of therapeutic TCRs while maintaining their target specificity and selectivity, researchers have turned to protein engineering solutions to create soluble therapies incorporating TCRs [24]. Tebentafusp, approved by the FDA in 2022, is part of a novel class of TCR-based therapeutics known as ImmTACs (Immune-mobilizing TCRs Against Cancer), which consists of a monovalent affinity-enhanced TCR fused to a CD3 scFv component. It redirects CD3+ T cells to a peptide derived from the melanoma-associated protein gp100 complexed with HLA*A02:01 [25]. Trials of tebentafusp demonstrated a survival benefit in metastatic uveal melanoma, a solid tumor with a low neoantigen burden that generally responds poorly to checkpoint inhibitors and chemotherapy [26,27]. Moreover, positive Phase 1 clinical efficacy and safety data were recently reported for brenetafusp, an ImmTAC molecule targeting PRAME/HLA-A*02 for the treatment of advanced cutaneous melanoma. A Phase 3 trial of brenetafusp is now underway [28]. These findings highlighted the potential of ImmTAC as an effective modality for targeting solid tumors, which largely remain elusive to innovative therapeutic modalities that have shown success in hematological malignancies.

### 2.4. TCRm Antibodies

**Figure 2:**
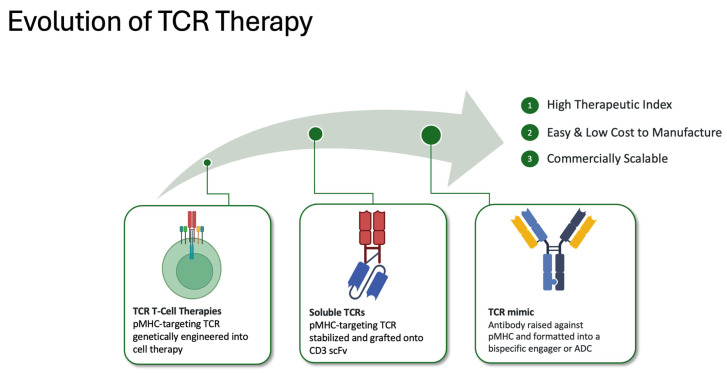
T-cell receptor technology has been leveraged in a variety of therapeutic modalities. Like other cell-based therapies, TCR T-cell therapies have a complex, costly manufacturing process that can be challenging to scale. Soluble TCR therapies overcome these manufacturing challenges but have limited binding affinity for pMHC targets. TCR-mimic antibody therapies unite the commercial and therapeutic strengths of antibodies but are not limited to extracellular protein targets.

TCR-like or TCR-mimic (TCRm) antibodies present an alternative route to harnessing the advantages of both TCRs and therapeutic antibodies while circumventing many challenges associated with traditional TCR-based agents (Figure 2). Like native TCR-containing agents, TCRms can recognize pMHC targets with high specificity and sensitivity but do so with the greater binding affinity of mAbs [29]. However, because naturally occurring antibodies typically lack TCR-like specificity for pMHC antigens, TCRm antibodies must be engineered or identified through target-driven in vitro screens.

The distinctive functional traits of TCRm antibodies make them a particularly promising modality for cancer immunotherapy. Firstly, because they recognize peptide fragments presented on the cell surface by MHC molecules, TCRms can be directed to a wider range of possible targets originating from intracellular tumor antigens, enabling interaction with previously “undruggable” targets [30]. Indeed, the majority of tumor-specific antigens related to cell growth, death, and proliferation are derived from intracellular proteins and thus cannot be targeted by traditional mAb therapies. The higher affinity of TCRm antibodies for pMHC targets offers a further advantage over TCR in oncology applications by reducing the risk of tumor immune cell evasion precipitated by low-affinity TCR binding [31]. Compared to TCR-based therapies, TCRm antibodies are easier to produce, making them more accessible for large-scale clinical applications.

The antitumor effects of TCRm antibodies can be mediated through a number of distinct mechanisms. As a standard antibody therapy, TCRms can initiate cytotoxicity indirectly through three broad pathways: antibody-dependent cell-mediated cytotoxicity (ADCC), complement-dependent cytotoxicity (CDC), and antibody-dependent cellular phagocytosis (ADCP) by macrophages [32]. TCRms can also exert direct cytotoxic effects in target cells via the activation of JNK and intrinsic caspase pathways, ultimately resulting in apoptosis [33]. Because patients are often immunocompromised, this direct mechanism is particularly notable in the context of cancer therapy as other immune cell-mediated cytotoxic mechanisms are functionally dependent on a largely intact immune system. TCRm antibody function can also be leveraged in the context of other immunotherapeutic modalities, such as antibody-drug conjugates or T-cell engagers, applications which will be discussed at greater length in a later section.

## 3. Application of TCRm Abs in Cancer Immunotherapy

Because naturally occurring antibodies typically lack TCR-like specificity for pMHC antigens and the pMHC is a distinctly complex antigen, the development of TCRm antibodies has traditionally been challenging. However, several key advances have helped researchers begin to overcome many of the persistent challenges in TCRm generation. Like conventional Abs, the two primary approaches to generating and isolating TCRms are immunization/hybridoma broad B cell technology and in vitro screening by phage display. The first reported TCRm antibody targeting a human tumor antigen, described by Chames and colleagues in 2000, was directed against a peptide encoded by the cancer-testis gene *MAGE-1* and complexed with HLA-A1 [34].

Especially in the context of cancer immunotherapy, target selection is a critical yet complex component of TCRm development. pMHC antigens can be grouped into three broad categories based on the origin of their associated peptide component: tumor-associated viral antigens, tumor-associated self-antigens (TAAs), and neoantigens [35]. TAAs are derived from normal proteins that are overexpressed in tumor cells, such as Wilms’ tumor 1 (WT1) or prostate-specific antigen (PSA) [36]. Neoantigens, or tumor-specific antigens (TSAs), result from genetic mutations occurring within cancer cells and are thus recognized as “non-self” by T cells when presented on the cell surface by MHC molecules [37]. While TSAs’ restriction to tumor cells makes them a promising target for immunotherapies sparing healthy cells, the highly patient-specific nature of these antigens often precludes their application in “off-the-shelf” therapies.

However, some mutations are shared among patients; for example, Hsiue et al. identified a TCRm antibody specific for the most common mutation in *TP53* and described successful preclinical application in a bispecific antibody, demonstrating possibilities for neoantigen-targeting TCRm therapeutics [38]. TAAs, on the other hand, are selectively overexpressed in tumor cells with limited expression in healthy cells but often fail to generate a robust immune response due to their “self”-associated origin [39]. TCRm antibodies have been successfully identified for multiple TAA targets, including PR1, WT1, PRAME, and others [40,41,42].

Epitope density is another key consideration in TCRm target selection, as an ideal pMHC target is densely expressed on tumor cells with minimal to no expression in healthy tissues. MHC-I expression is often downregulated in cancer cells, presenting a challenge to this goal. However, recent research has demonstrated the cytotoxic efficacy of TCRm bispecific antibodies targeting peptide-HLA complexes derived from mutated RAS with as few as three copies per cell [43]. Because most TCRm bind only a few target residues within the pMHC, there is a theoretical potential for cross-reactivity with off-target epitopes sharing these residues at key contact positions; however, the fact that these potentially cross-reactive sequences must be processed and presented on the cell surface in a highly specific conformation reduces the likelihood of this issue [44]. The complex nature of the pMHC target necessitates the availability of plentiful and high-quality recombinant pMHC molecules for antibody generation. Recent advances have simplified recombinant pMHC generation, including the development of conformationally “open” MHC-I molecules that enable rapid, stable peptide loading for TCRm antibody characterization across multiple HLA types [45]. Additionally, an increased understanding of the role of TCRm structure in binding affinity has led to a more focused Ab isolation approach that re-engineers Abs to engage pMHC in a conformationally “TCR-like” manner [46].

## 4. Progress in Preclinical and Clinical Development of Therapeutic TCRm Antibodies

Preclinical development of TCRm antibodies over the last two decades has highlighted viable solutions to obstacles in this modality and underscored their vast potential for immunotherapeutic applications. Specificity for HLA subtypes is a persistent challenge in TCRm development, but some findings demonstrate the potential for engineering TCRm antibodies that overcome HLA subtype restriction. For example, Ataie and colleagues hypothesized that the structure of ESK1, an identified TCRm specific for the WT1-derived RMF peptide in complex with HLA-A*02:01, would accommodate binding with other HLA-A*02 subtypes. Their findings confirmed that ESK1 could bind with high affinity to six HLA-A*02 subtypes, illustrating the potential for TCRm-based immunotherapies with wider patient compatibility [47]. Identifying TCRm with the desired affinity and specificity remains the primary hurdle; beyond that, many of the established strategies used to optimize mAbs can be applied to increase the potency and efficacy of TCRm therapeutics.

Because of structural differences in their engagement with pMHCs, TCRms require higher binding affinities than TCRs to overcome the typically low density of antigen targets and exert sufficient therapeutic potency. A study by Zhao and colleagues demonstrated that affinity maturation via mutagenesis and yeast display enhanced binding affinity of a TCRm targeting WT1-HLA-A2 100-fold [48]. Researchers have also identified various engineering approaches to enhance the antitumor efficacy of TCRms and optimize pharmacokinetic properties to maximize therapeutic exposure in circulation [49]. Integrating TCRm and TCRm-based technology into other therapeutic modalities, such as T-cell engagers and antibody-drug conjugates, may provide ideal formats for maximizing their potential in oncology.

A growing body of research has demonstrated that re-engineering TCRm antibody components into other therapeutic formats can enhance antitumor efficacy while maximizing their safety and viability in a clinical context. For example, CARs derived from TCRms can be leveraged to generate CAR-T cells capable of targeting intracellular antigens [50]. The first preclinical example of a TCRm CAR was a WT1-HLA-A*02:01-specific CAR-T cell therapy reported in 2014 by Rafiq and colleagues [51]. Other preclinical and clinical examples have been developed, including a scFv-CAR-T cell therapy targeting alpha-fetoprotein (AFP), currently in Phase I/II trials for the treatment of pediatric hepatoblastoma [52]. While TCRm CAR-Ts are a promising modality, their patient-specific nature confers the same clinical development and production challenges faced by other autologous cell therapies.

The first TCRm T-cell engaging bispecific Ab RO7283420 has been evaluated in acute myeloid leukemia (AML) targeting HLA-A2/WT1. The preclinical evaluation of RO7283420 in in vivo humanized AML xenografts and ex vivo AML co-culture models showed strong T-cell-mediated AML cell killing [53]. Recently reported phase I trial results revealed pharmacodynamic evidence of T-cell activation and expansion, in line with the expected mechanism of TCRm T-cell engaging BsAbs, but only modest clinical activity [54].

While we have seen great progress on TCRm in clinical studies for liquid cancer, solid tumors pose a particular challenge due to their heterogeneous antigen presentation, which can vary between tumor cells and even within the same tumor. However, recent data at preclinical stage indicate that TCRm antibodies can still be effective in these settings. For instance, Liu et al. demonstrated the use of TCRm antibodies in a solid tumor model in treatment of hepatocellular carcinoma, showing effective targeting and reduction in tumor volume [55]. The key to overcoming the heterogeneity appears to lie in the careful selection of peptide-MHC targets that are more uniformly presented across tumor cells, as well as the design of TCRm antibodies with broader but still specific binding profiles.

### 4.1. Engineering Strategies for T-Cell Redirecting Antibody Therapeutics

TCRm technology can also be applied in bispecific T-cell redirecting therapeutics by uniting target-specific scFvs with a CD3-binding scFv. A 2015 study by Dao and colleagues described a bispecific therapeutic antibody derived from the TCRm ESK1, which targets a pHLA complex of WT1. Despite the low density of target complexes on the cell surface, the ESK1-BiTE successfully induced a robust cytolytic T-cell response toward leukemic and tumor cells in vitro and in mice [56].

Further exploration of BsAbs, including TCRm antibodies, has elucidated the importance of protein geometry in modulating binding affinity and antitumor efficacy, enabling precise geometry engineering to optimize their performance [57]. For example, preclinical studies of a FcRH5/CD3 BsAb for multiple myeloma demonstrated activation of intracellular T cell signaling and clarified the role of target size and epitope location in efficient synapse formation and resulting T cell activation [58].

Huber and colleagues reported that CD3 binding affinity also has important implications for the T cell activation and potential toxicity of CD3+ T cell redirecting BsAbs [59]. This finding provides important new considerations for the design of TCRm BsAbs outside of TAA/TSA specificity, as CD3 affinity tuning could provide an additional pathway to minimizing toxicities such as cytokine release syndrome (CRS) while maximizing therapeutic efficacy.

Removal of the Fc region is another engineering strategy to minimize adverse immune effects for antibody therapeutics; however, this creates an additional limitation in therapeutic exposure by removing Fc-driven cellular recycling via the neonatal Fc receptor (FcRn). Blinatumomab, the first US-approved BiTE, requires continuous intravenous infusion for up to four weeks per treatment cycle to maintain sufficient concentration in circulation.

Alternatively, generating BiTE molecules with engineered FcRn binding affinities is another strategy to increase the half-life of bispecifics while maintaining optimal safety profiles. Suurs et al. demonstrated that fusion of an Fc domain to a mesothelin-targeted BiTE extended half-life as well as tumor uptake [60]. Fusion of human albumin sequences engineered with FcRn of varying binding affinities with a BiTE-like CD3 TCE also enabled fine-tuning of half-life extension, providing a more tailored approach to pharmacokinetic optimization of CD3-redirecting bispecifics [61].

A further consideration for the mechanism of action in TCRm TCEs is the need to overcome the hostile and immunosuppressive tumor microenvironment for effective antitumor activity. Solid tumors are surrounded by stromal cells that suppress anti-tumor immunity, including cancer-associated fibroblasts (CAFs), T_REG_ cells, tumor-associated macrophages (TAMs), and myeloid-derived suppressor cells, as well as immunosuppressive cytokines and soluble factors. This hostile Tumor Microenvironment (TME) limits the efficacy of endogenous cytotoxic T-cells in protecting against tumor development. Disrupting these mechanisms via targeted destruction of CAFs and TAMs is one promising avenue to increase solid tumor penetration and enhance the antitumor efficacy of T-cell-engaging bispecific antibody therapeutics [62]. Other efforts in the field of cancer immunotherapy are likely to involve identifying optimal combination therapies with TCRm bispecific T-cell redirectors that enhance anti-tumor immunity while minimizing toxicity [63].

### 4.2. Incorporating TCRm Technology in Antibody-Drug Conjugates

While antibody-drug conjugates (ADCs) are another potential modality for leveraging the targeting potential of TCRms, they have received less attention than T-cell redirecting compounds, due in part to concerns over low pMHC target density. Target peptide-HLA complexes are typically expressed on the tumor cell surface in quantities below the threshold for conventional surface antigens to internalize cytotoxic payloads and kill tumor cells [64]. TCRm-containing ADCs with substantial antitumor efficacy were first reported over twenty years ago. Some of the earliest examples targeted pHLA complexes derived from melanoma-associated gp100 or MART-1 with antibody fragments linked to a bacterial toxin [65,66]. While these initial ADCs yielded meaningful in vivo antitumor effects for high-density pHLA targets, their results were far less robust for tumors expressing fewer than 10,000 pHLA copies per cell. More recent studies have supported strategies to increase cytotoxic potency to overcome this limitation. For example, Shen and colleagues described a bispecific TCRm ADC targeting two WT1 epitopes simultaneously that demonstrated greater antitumor efficacy than ADCs targeting each epitope individually [67].

ADCs are a promising drug class for delivering cytotoxic payloads to specific cells, but their clinical application as oncology therapies can be limited by the expression of TAAs in normal cells, causing off-target toxicity. Because of this, tumor-specific neoantigens (TSNAs) are a more viable target for ADC therapies that minimize harm to healthy cells. For example, the KRAS G12V mutation is implicated in multiple cancers. Shen and colleagues generated two TCRm ADC candidates successfully targeting KRAS G12V/HLA-A*0201, achieving specific anti-tumor activity in vitro and in vivo without obvious off-target toxicity [68]. These findings validated the strategy of developing TCRm ADCs against intracellular TSAs. Continued exploration of TAA versus TSA targets for TCRm ADCs will be essential for identifying candidate compounds that minimize impact on healthy tissues while maximizing clinical viability. Additionally, elements outside of the target selection, such as the choice of cytotoxic payload and the linker that joins ADC components, are also key in optimizing pharmacokinetic performance and tolerability [69]. Continued advances in payload, drug-to-antibody ratio (DAR), and linker technology, as well as the identification of common TSAs within and across cancers, will drive innovation in clinically viable TCRm ADCs.

There are a variety of TCRm candidates currently under preclinical and early-phase clinical development, representing ADCs, TCEs, TCRm-T cells, and IgGs (Table 1). While the majority target germline variants and tumor-specific neoantigens for the treatment of cancer, several utilize TCRm modalities to address viral targets, including HIV and Hepatitis B.

## 5. Future Directions in TCRm Cancer Immunotherapy

TCR mimic antibodies represent a relatively new yet highly promising class of tools in the development of cancer immunotherapies. Their capacity to recognize intracellular peptide targets with high specificity and affinity unlocks a much broader range of novel druggable targets. Despite substantial progress in TCRm discovery and engineering, challenges remain in bringing successful TCRm-based cancer immunotherapies to the clinic.

The HLA restriction of TCR-mimic antibodies also hinders their wider application in immunotherapeutic modalities, as each identified TCRm is only compatible with a specific population of patients. Because HLA-A*02 is a common allele in many populations, many cancer-associated antigens associated with this subtype have been identified and leveraged in TCRm discovery. To maximize clinical applicability, TCRm discovery can be broaden to include alleles prevalent in specific ethnic groups, such as HLA-A*24:02, a major HLA allele that covers about 36% of the Japanese patient population [79]. However, as efforts to engineer TCRms for wider HLA compatibility have had some success within multiple HLA-A*02 serotypes, it is feasible that a deeper understanding of the pMHC structure and interaction dynamics with TCRms could yield “universal” TCRm-based therapeutics targeting specific TAAs/TSAs without restriction to an HLA subtype [47].

**Figure 3:**
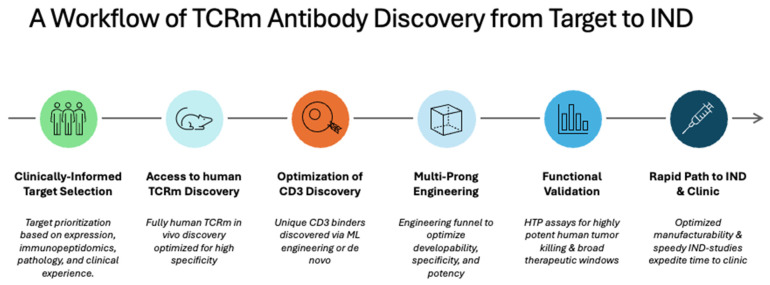
The emerging workflow for TCRm T-cell redirector discovery and development, from target selection to IND, involves starting with a clinically relevant target. The State-of-the-Art human antibody discovery and specificity testing are applied to obtain highly specific TCRm binders. These binders are then engineered with an optimized CD3 antibody to enhance potency while minimizing safety risks, such as cytokine release syndrome (CRS).

As more powerful TAA and TSA therapeutic targets are identified, quality pMHC antigen production will be foundational to delivering on the potential of TCRm antibody therapeutics. By uniting selection, engineering, and validation capabilities into an end-to-end, integrated workflow, researchers can better identify high-specificity, high-affinity, human TCRm antibodies from a comprehensive range of potential targets (Figure 3). As conceptualized, this workflow enables the production and use of high-quality pMHC immunogens for in vitro, in vivo, and in silico antibody discovery. Crystal structure studies of TCR cross-reactivity have informed our understanding of how TCRms targeting the same antigen differ in interaction with off-target peptides [80]. Antibody leads can be selected through a series of specificity assays to minimize off-target non-specificity effects, ensuring that the most viable candidates can proceed to preclinical development. The relevancy of candidates can be further validated through assays of key functional traits, such as specificity and high-affinity binding of TCRms. This approach is ideal for therapeutic targets with more understood, well-defined biology and readily available functionally relevant assays with the screening reagents already accessible. Identified TCRm-specific binders can then be optimized into viable therapeutic modalities such as bispecific T-cell engagers or ADCs to potentiate their function in targeting tumors.

While most TCRm therapeutics in development are aimed at cancer treatment, there are potential opportunities for TCRms to target pathogenic T cells for the treatment of autoimmune disease. A 2020 review by Davidson and Zhang summarized studies using a mAb targeting a key pathogenic epitope from insulin to treat a spontaneous mouse model of T1D and discussed the translational potential of therapies based on this approach [81].

In addition to using antibody blocking and depletion of pHLA targets, TCR-targeted antibodies could be leveraged to eliminate pathogenic T cells. A recent interesting example of this application was a documented single-patient treatment for ankylosing spondylitis using antibodies targeting a disease-associated TRBV-9 TCR epitope presented by HLA-B*27. After repeated administration of this TRBV-9+ T cell elimination therapy, the patient achieved complete remission within three months, which persisted for four years with continued periodic treatment [82]. This finding demonstrates the potential of selective T-cell depletion as a possible curative therapy for autoimmune disease.

While obstacles remain in generating safe and effective TCRm-based cancer immunotherapies, innovations and improvements in antibody discovery, selection, and development methods, such as these, are key building blocks to advancing this goal. As researchers and drug developers continue to identify promising antigen targets, deepen our understanding of TCRm structure and function, and establish optimal strategies for engineering TCRms into therapeutic modalities, the field makes increasing progress in delivering on the promise and power of immunotherapy to fight cancer.

## 6. Conclusions

TCRm can be engineered with a number of additional mechanisms of actions (MoA) to tackle disease. When TCRm is engineered with an additional arm binding to T cells (bispecific T-cell engager), TCRm antibodies redirect T cells to kill target cells, facilitating direct cytotoxicity. When TCRm are attached to toxic or radioisotopes payloads, such as an ADCs or RLT, they deliver cell-killing agents to target cells with high specificity, sparing healthy tissues. Together, these antibody-based strategies open new avenues for untreatable diseases.

## Figures and Tables

**Table 1:** List of recent TCRm therapeutics in preclinical and clinical development.

Target/HLA	Modality	Stage	Sponsor	Reference
WT1/A2	TCE bispecific	Phase 1	Roche	[54]
AFP/A2	TCRm-T	Phase 1	Eureka	Clinicaltirals.gov (NCT03998033)
PR1/A2	IgG1	Phase 1	MD Anderson Cancer Center	[70]
MAGE-A4/A2	TCE bispecific	Phase 1	CDR-Life	Clinicaltrials.gov (NCT06402201)
MAGE-A4/A2	TCE bispecific	Phase 1	Genentech	Clinicaltrials.gov (NCT06372574)
survivin-2B80-88/A24	TCE bispecific	Preclinical	U. Toyama	[71]
KRAS G12V	ADC	Preclinical	Huabo Biopharm Co., Ltd.	[68]
WT1C/A2	IgG	Preclinical	U. Toyama	[41]
HIV/A2	TCE bispecific	Preclinical	Johns Hopkins University	[72]
pIRS2/ A2	TCE bispecific	Preclinical	Memorial Sloan Kettering Cancer Center	[73]
HPV-16E7/A2	TCE bispecific	Preclinical	Memorial Sloan Kettering Cancer Center	[74]
NDC80/A2	TCRm-CAR-T	Preclinical	Eureka/MSKCC	[75]
KRAS G12V/A3	TCE bispecific	Preclinical	Johns Hopkins University	[76]
MART1/A2	TCE bispecific	Preclinical	Stanford University	[46]
CG1/A2	TCE bispecific	Preclinical	Crossbow Therapeutics	[77]
HBV/A2	TCE bispecific	Preclinical	Xiamen University	[78]
